# Simultaneous determination of twelve mycotoxins in edible oil, soy sauce and bean sauce by PRiME HLB solid phase extraction combined with HPLC-Orbitrap HRMS

**DOI:** 10.3389/fnut.2022.1001671

**Published:** 2022-09-28

**Authors:** Donghui Luo, Jingjing Guan, Hao Dong, Jin Chen, Ming Liang, Chunxia Zhou, Yanping Xian, Xiaofei Xu

**Affiliations:** ^1^College of Food Science and Engineering, Guangdong Ocean University, Yangjiang, China; ^2^Chaozhou Branch of Chemistry and Chemical Engineering Guangdong Laboratory (Hanjiang Laboratory), Chaozhou, China; ^3^Key Laboratory of Green Processing and Intelligent Manufacturing of Lingnan Specialty Food, Guangdong Provincial Key Laboratory of Lingnan Specialty Food Science and Technology, College of Light Industry and Food Sciences, Ministry of Agriculture, Zhongkai University of Agriculture and Engineering, Guangzhou, China; ^4^Guangzhou Quality Supervision and Testing Institute, Guangzhou, China

**Keywords:** PRiME HLB, HPLC-Orbitrap HRMS, edible oil, mycotoxin, soy sauce

## Abstract

A solid phase extraction-high-performance liquid chromatography-tandem Orbitrap high resolution mass spectrometry (HPLC-Orbitrap HRMS) method was established for the determination of 12 mycotoxins (ochratoxin A, ochratoxin B, aflatoxin B1, aflatoxin B2, aflatoxin G1, aflatoxin G2, HT-2 toxin, sterigmatocystin, diacetoxysciroenol, penicillic acid, mycophenolic acid, and citreoviridin) in edible oil, soy sauce, and bean sauce. Samples were extracted by 80:20 (*v:v*) acetonitrile-water solution, purified by PRiME HLB column, separated by aQ C18 column with mobile phase consisting of 0.5 mmol/L ammonium acetate-0.1% formic acid aqueous solution and methanol. The results showed that the limits of detection (LODs) and limits of quantification (LOQs) of 12 mycotoxins were 0.12–1.2 μg/L and 0.40–4.0 μg/L, respectively. The determination coefficients of 12 mycotoxins in the range of 0.20–100 μg/L were > 0.998. The average recoveries in soy sauce and bean sauce were 78.4–106.8%, and the relative standard deviations (RSDs) were 1.2–9.7% under three levels, including LOQ, 2× LOQ and 10 × LOQ. The average recoveries in edible oil were 78.3–115.6%, and the precision RSD (*n* = 6) was 0.9–8.6%. A total of 24 edible oils, soy sauce and bean sauce samples were analyzed by this method. AFB1, AFB2, sterigmatocystin and mycophenolic acid were detected in several samples at concentrations ranging from 1.0 to 22.1 μg/kg. The method is simple, sensitive, and rapid and can be used for screening and quantitative analysis of mycotoxin contamination in edible oil, soy sauce, and bean sauce.

## Introduction

Mycotoxins are toxic secondary metabolites produced by mycotoxin-producing fungi under suitable environmental conditions (1–3). Currently, there are more than 400 known mycotoxins. Edible oils such as rapeseed oil, peanut oil, corn oil, sesame oil, soy sauce and bean sauce are easily contaminated by mycotoxins because most cereals are used as raw materials in the preparation process (4, 5). It has been reported that ~25% of wheat, corn, sorghum and rice produce toxic and harmful mycotoxins due to mildew during production, processing, transportation and storage every year (6). At present, aflatoxin (AFT), ochratoxin A (OTA) and zearalenone (ZEN) have a great influence on human health, which will damage human liver function, cause cancer and teratogenicity and induce immunosuppressive diseases after exceeding a certain intake (7, 8). The World Health Organization has included mycotoxins in the key monitoring objects of the food safety system (9). In China, GB2761-2017 “National Standard for Food Safety Limits of Mycotoxins in Food” also has strict regulations on the limit indicators of some mycotoxins in food. Under natural conditions, edible oils and condiments may be contaminated by various mycotoxins. According to the current national standards, it is necessary to use several detection methods for multiple experimental analyses to determine the content of different mycotoxins (10–12). The determination methods are not only cumbersome but also inefficient. Therefore, it is urgent to establish a synchronous detection method for multiple mycotoxins.

Anastassiades et al. (13) proposed the QuEChERS (Quick, Easy, Cheap, Effective, Rugged, Safe) method, namely, dispersive solid phase extraction, but it is still insufficient to extract many toxins from complex substrates. Oasis PRiME HLB is a type of reversed-solid phase extraction (SPE) adsorbent that can simplify and accelerate the SPE process and can obtain cleaner extracts compared with other sample pretreatment methods. Compared with other SPE products, it can also remove more than 90% of endogenous phospholipids and is widely used in the detection of food organic pollutants (14). At present, colloidal gold immunochromatography, enzyme-linked immunosorbent assay (ELISA) kits, immunoaffinity column-high-performance liquid chromatography and isotope dilution liquid chromatography tandem mass spectrometry are commonly used to detect mycotoxins (3, 15, 16). Liquid chromatography-tandem mass spectrometry (LC–MS/MS) has higher selectivity and sensitivity and has gradually become the main means for the simultaneous detection of various mycotoxins (9, 17, 18). Orbitrap high-resolution mass spectrometry (Orbitrap HRMS) has higher selectivity and resolution than ordinary mass spectrometry and can effectively reduce the interference of impurities in complex matrices (19–22).

Therefore, the samples were simply extracted and purified by a PRiME HLB solid phase extraction column in this study, and the conditions of liquid chromatography and mass spectrometry were optimized. An HPLC-Orbitrap HRMS method for the determination of mycotoxins in edible oil, soy sauce, and bean sauce was established. The method has advantages such as simplicity, rapidity and high flux, which is suitable for the screening and detection of mycotoxins in edible oil, soy sauce and bean sauce and reduce food safety problems caused by mycotoxin residues.

## Materials and methods

### Instruments and reagents

The Thermo Q Exactive Focus High Performance Liquid Chromatography–Mass Spectrometry System includes a Dionex Ultimate 3000 Liquid Phase Pump, Autosampler, Column Oven and Orbitrap High Resolution Mass Spectrometry Section (Thermo Fisher Scientific, Massachusetts, USA). XCalibur 4.0 software (Thermo Fisher Scientific, Massachusetts, USA) was used for mass spectrometer control and data processing. A Thermo Accucore aQ C18 (2.1 × 150 mm, 2.6 μm) was used as the chromatographic column. The samples were vortexed with an MS3 basic vortex mixer (IKA GmbH, Staufen, Germany). A KQ-250DV CNC ultrasonic cleaning device (Kunshan, Jiangsu, China) was used for supersonic-assisted extraction. Ultrapure water (18.2 MΩ·cm) prepared from a Milli-Q ultrapure system (Millipore, Bedford, MA, USA) was used in the whole experiment.

Twelve mycotoxins, as shown in Table 1, were purchased from Shanghai Anpu Scientific Instruments Co., Ltd. and Adamas Reagent Company of Switzerland. These standards all have a purity of or higher than 98.0%. Acetonitrile, methanol, ammonium acetate, and formic acid were obtained from CNW Technologies GmbH (CNW, Düsseldorf, Germany). A PRiME HLB solid phase extraction column was purchased from Waters Company (60 mg/3 cc, Waters, Beverly, MA, USA).

**Table 1:** The gradient elution procedure of HPLC.

**Time (min)**	**Mobile phase**
0–2.0	90%A^*^
2.0–3.0	90%A-80%A
3.0–5.0	80%A-74%A
5.0-7.0	74%A
7.0–10.5	74%A-40%A
10.5–13.5	40%A
13.5–14.5	40%A-5%A
14.5–17.0	5%A
17.0–18.0	5%A-90%A
18.0–20.0	90%A

* The mobile phase consists of 0.5 mmol/L ammonium acetate solution containing 0.1% formic acid (A) and methanol (B).

Preparation of standard solutions: The standard reference materials of each mycotoxin were carefully measured and prepared into a 1.00 mg/L mixed standard reserve solution with acetonitrile, which was stored in a refrigerator at 4 °C. Then, an appropriate amount of standard reserve solution was transferred and prepared with acetonitrile to form a series of mixed standard curves with concentrations of 100, 50, 20, 10, 5, 2, 1, 0.5, and 0.2 μg/L.

### Sample preparation

Thirteen kinds of edible oil samples and 11 soy sauce and bean sauce samples were purchased from local supermarkets and online shopping malls in Guangzhou. A 2.0 g sample was weighed and placed in a 50 mL centrifuge tube, and 20 mL acetonitrile-water 80:20 (*v/v*) solution was added. After mixing, the sample was extracted by oscillation for 10 min and centrifuged at 500 × g for 5 min at room temperature. Ten milliliters of supernatant was transferred into a 50 mL centrifuge tube, and 10 mL of n-hexane was added and vortexed for 1 min. Then, the mixture was centrifuged at 500 × g for 3 min at room temperature. After that, a PRiME HLB column and HLB solid phase extraction column were adopted for purification. Finally, the purified solution was dried with nitrogen at 40°C. Then, the residual was reconstructed with 1.0 mL acetonitrile and filtered by a 0.22 μm polytetrafluoroethene (PTFE) syringe filter (Waters, Beverly, MA, USA).

### LC-Orbitrap HRMS conditions

The mobile phase consisted of 0.5 mmol/L ammonium acetate solution containing 0.1% formic acid (A) and methanol (B). The gradient elution procedure is presented in Table 1. The injection volume was 5 μL. The flow rate was 0.3 mL/min.

#### Mass spectrometry conditions

The Q Exactive Focus mass spectrometry system was equipped with a HESI ion source using positive ion mode, spray voltage 3.5 kV, and capillary and spray temperatures of 320°C and 250°C, respectively. The sheath and auxiliary gas pressures were set at 45 arb and 8 arb, respectively, and the S-lens RF voltage was 50 V. Both the spray gas and collision gas were nitrogen. Correction solutions (a solution of 2 μg/mL caffeine, 1 μg/mL MRFA, 0.001% Ultramark 1621 and 0.0005% n-butylamine; a solution of 2.9 μg/mL sodium dodecyl sulfate, 5.4 μg/mL sodium taurocholate and 0.001% Ultramark 1621) were used to correct the mass axis once every 7 days. The scanning mode was full MS/dd-MS2 mode. The full MS first-level full scanning range was *m/z* 100–650, resolution was 70000, automatic gain control AGC and automatic injection time IT were set to 1.0 e^6^ and 100 ms, respectively; the data-dependent AGC of dd-MS2 was set to 1.0 e^5^, the resolution was set to 17,500, the maximum IT was set to 60 ms, the separation window was set to 2.0 *m/z*, the normalized collision energy (NCE) of each compound was set to 20, 40, and 60%, and the dynamic exclusion was set to 8 s.

## Results and discussion

### Optimization of mass spectrometry conditions

Compared with triple quadrupole mass spectrometry, Orbitrap high-resolution mass spectrometry is simpler to operate and optimize mass spectrometry conditions (20). First, 12 target standards (concentration 100 μg/L) were scanned by full MS with a resolution of 70,000, and qualitative screening and quantitative detection were carried out according to the accurate mass number of primary parent ions of the target compounds. The quasi-molecular ion peaks of [M + H] ^+^, [M + NH_4_] ^+^, and [M + Na] ^+^ may be produced in the positive ion mode, and the quasi-molecular ion peaks of [M-H]^−^ are mainly produced in the negative ion mode. By comparing the response values of each quasi-molecular ion peak in the two modes, it was found that aflatoxin could produce molecular ions in both positive and negative ion modes, but the response value of [M-H]^−^ was far lower than that of [M + H] ^+^; HT-2 toxin belongs to trichothecenes, and its parent ion can form [M + H] ^+^, [M + Na] ^+^ and [M + NH_4_] ^+^, but the conjugate of [M + Na] ^+^ has the highest response value and sensitivity, so HT-2 toxin chooses [M + Na] ^+^ as the parent ion. Diacetoxysciroenol can form [M + Na] ^+^ and [M + NH_4_] ^+^, but the response value and sensitivity of [M + Na] ^+^ are higher; the other six toxins all have [M + H] ^+^ ions in positive ion mode, so positive ion scanning mode was used for detection in this experiment. Then, full MS/dd-MS_2_ mode was adopted, in which dd-MS_2_ was used as the confirmation mode. When the parent ion strength reached the set threshold (1 × 10^6^), it automatically triggered secondary mass spectrometry scanning, and the information of secondary fragment ions could be further confirmed by combining the accurate mass number and retention time of primary parent ions. The precise mass number, mass accuracy error and retention time of the 12 mycotoxins are shown in Table 2.

**Table 2:** HPLC and Orbitrap HRMS parameters of 12 mycotoxins.

**Compounds (abbreviations)**	**Molecular formula**	**Ion**	**Rt/min**	**Measured *m/z***	**Theoretical *m/z***	**Fragment ions**	**Error (ppm)**	**NCE (%)**
Ochratoxin A (OTA)	C_20_H_18_ClNO_6_	[M+H]^+^	13.44	404.08789	404.08954	358.08279, 257.02112	−4.08	10
Ochratoxin B (OTB)	C_20_H_19_NO_6_	[M+H]^+^	12.39	370.12781	370.12851	223.05954, 324.12219	−1.89	10
Aflatoxin B1 (AFB1)	C_17_H_12_O_6_	[M+H]^+^	11.41	313.06979	313.07066	285.07471, 270.05087	−2.78	50
Aflatoxin B2 (AFB2)	C_17_H_14_O_6_	[M+H]^+^	11.19	315.08524	315.08631	287.09055, 259.05942	−3.40	50
Aflatoxin G1 (AFG1)	C_17_H_12_O_7_	[M+H]^+^	10.76	329.06464	329.06558	243.06461, 215.6949	−2.86	50
Aflatoxin G2 (AFG2)	C_17_H_14_O_7_	[M+H]^+^	10.48	331.08035	331.08123	313.06967, 245.08006	−2.66	50
HT-2 toxin (HT-2)	C_22_H_32_O_8_	[M+Na]^+^	11.74	447.19690	447.19894	149.02306, 345.12979	−4.56	40
Sterigmatocystin (ST)	C_18_H_12_O_6_	[M+H]^+^	15.39	325.07004	325.07066	281.04382, 310.04620	−1.91	40
Diacetoxyscirpenol (DAS)	C_19_H_26_O_7_	[M+Na]^+^	10.70	389.15549	389.15707	89.05946, 133.08578	−4.06	50
Penicillic acid (PA)	C_8_H_10_O_4_	[M+H]^+^	4.92	171.06497	171.06518	125.05949, 72.04420	−1.23	40
Mycophenolic acid (MA)	C_17_H_20_O_6_	[M+H]^+^	12.30	321.13220	321.13326	207.06477, 159.04353	−3.30	40
Citreoviridin (CIT)	C_23_H_30_O_6_	[M+H]^+^	14.29	403.21017	403.21150	139.03871, 83.04883	−3.30	40

### Optimization of chromatographic conditions

**Figure 1:**
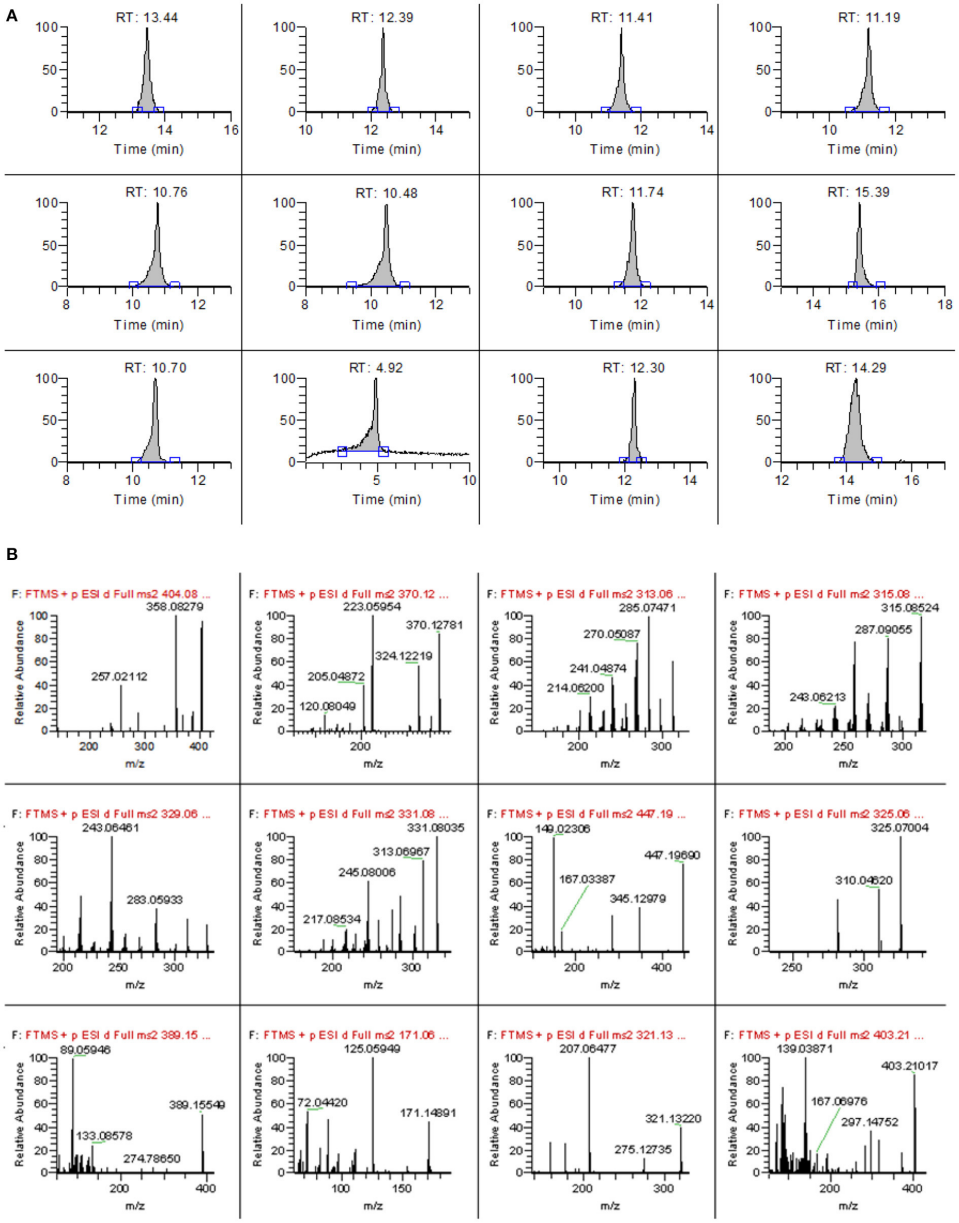
The chromatograms **(A)** and high-resolution mass spectrum **(B)** of 12 mycotoxins.

The type and proportion of the mobile phase not only affect the retention time and peak shape of the target compound but also affect the ionization efficiency of the target compounds, thus affecting the sensitivity (23–25). In this study, four different mobile phases, A: 0.1% formic acid water-methanol, B: 1 mmol/L ammonium acetate-0.1% formic acid water-methanol, A: 0.1% formic acid water-acetonitrile, and D: 1 mmol/L ammonium acetate-0.1% formic acid water-acetonitrile, were compared on the mass spectral responses of 12 mycotoxins. The results showed that when A was the mobile phase, all toxins had a mass spectrometry response. However, when ammonium acetate is present in the mobile phase, the ionic response of each target substance is obviously enhanced. Therefore, the effects of different concentrations of ammonium acetate (0.1, 0.5, 1, 2, and 5 mmol/L) on the response intensity of mass spectrometry were further optimized. The results showed that when the concentration of ammonium acetate increased to 0.5 mmol/L, the ionic responses of most of the target compounds were enhanced, but the concentration of ammonium acetate continued to increase, but the response values of the other eight target compounds decreased slightly except for four aflatoxins. Therefore, 0.5 mmol/L ammonium acetate-0.1% formic acid aqueous solution was finally selected as the mobile phase. The main organic phases were acetonitrile and methanol. It was found that the peak shape of some target compounds was poor when acetonitrile was used as the mobile phase, so methanol was selected as the organic phase in this study. Therefore, 0.5 mmol/L ammonium acetate-0.1% formic acid aqueous solution-methanol was used as the mobile phase. The chromatographic and mass spectra of 12 mycotoxins after optimization are shown in Figures 1A,B.

### Optimization of pretreatment

#### Optimization of extraction solvent

**Figure 2:**
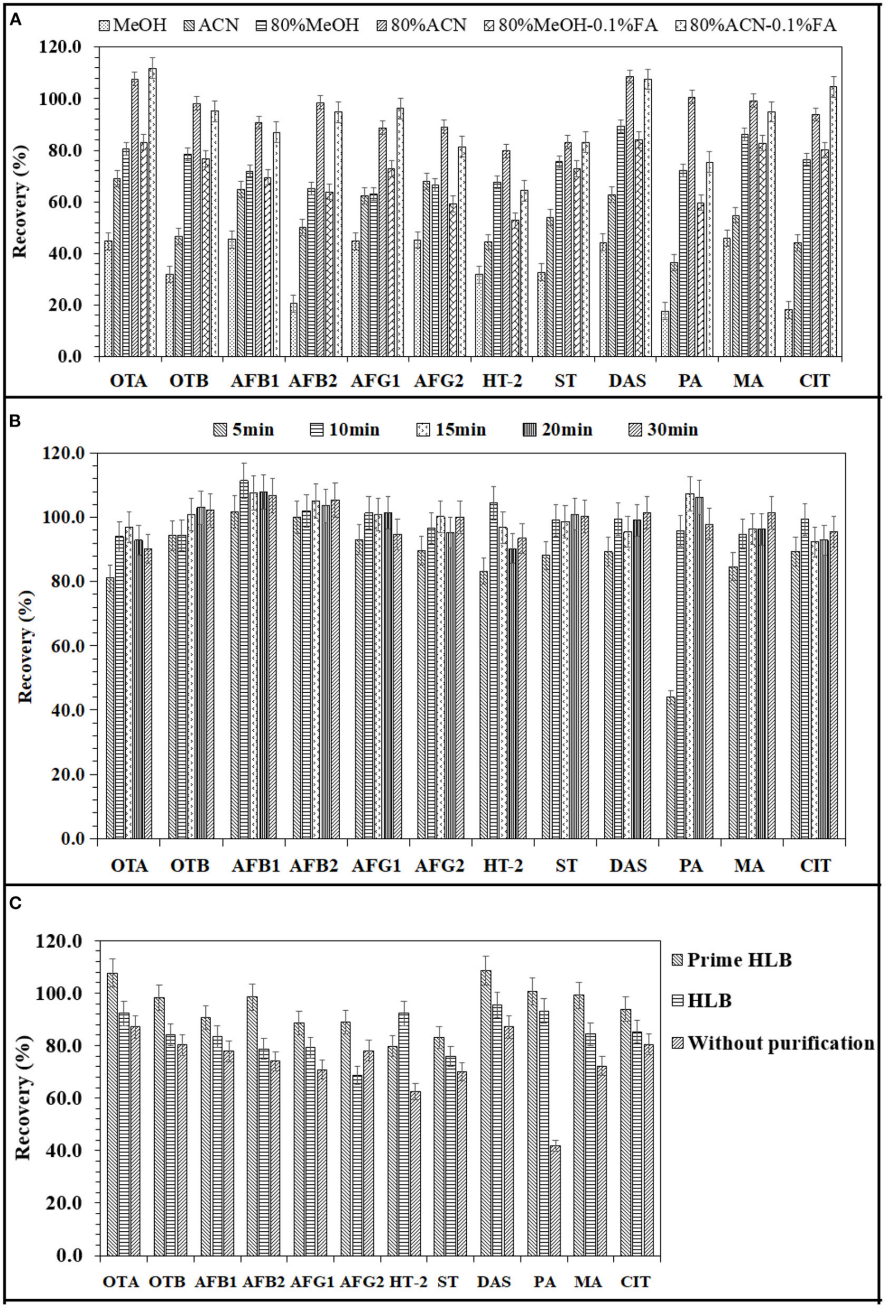
Effects of extraction solvent **(A)**, extraction time **(B)**, and various solid phase extraction columns **(C)** on the extraction recoveries of 12 mycotoxins.

Mycotoxins were mainly extracted by methanol, acetonitrile or a mixture of these two solvents and water in different proportions (26, 27). Therefore, this study compared the extraction efficiency of 12 mycotoxins from soy sauce by six insoluble solvent systems: methanol, acetonitrile, 80% methanol-water, 80% acetonitrile-water, 80% methanol-water-0.1% FA and 80% acetonitrile-water-0.1% FA. The results are shown in Figure 2A. The extraction effect of acetonitrile on 12 mycotoxins was significantly better than that of methanol. The addition of water can significantly improve the extraction rate. Eighty percent acetonitrile-water and 80% acetonitrile-hydr-0.1% FA had little effect on the mycotoxin extraction efficiency, except for HT-2 and penicillin. However, 80% acetonitrile-water was better than 80% acetonitrile-hydr-0.1% FA in the extraction efficiency of HT-2 and penicillic acid. Hence, 80% acetonitrile-water was finally selected as the extraction solvent.

#### Optimization of extraction time

In this study, 80% acetonitrile-water was used as the extraction solvent, and the effects of different shaking extraction times (5, 10, 15, 20, and 30 min) on the extraction of 12 mycotoxins from condiments were compared. The results are shown in Figure 2B. With increasing extraction time, the recovery rate gradually increased, and after increasing to 10 min, the recovery rate remained basically unchanged. Therefore, a 10 min shaking time was finally selected as the extraction time.

#### Optimization of purification conditions

The effects of two different solid phase extraction columns (HLB column and PRiME HLB column) on the recovery of mycotoxins were investigated. The HLB column was a reversed-phase solid phase extraction column, and the impurities were removed by leaching after adsorbing the target substance. Compared with other SPE products, it can remove 95% of common matrix interfering substances (such as phospholipids, fats, salts and proteins) (28). As shown in Figure 2C, the recoveries of 12 mycotoxins were improved by two purification columns. After PRiME HLB purification, the recoveries of mycotoxins ranged from 79.8 to 108.6%, and those obtained by HLB column purification ranged from 68.8 to 93.1%. Without purification, the recoveries of 12 mycotoxins ranged from 41.9 to 87.2%. Compared with the two kinds of solid phase extraction columns, the recovery rate of the impurity adsorption solid phase extraction column was better than that of the target substance adsorption solid phase extraction column except for HT-2 toxin, mainly because the HLB column did not have specific adsorption for a specific toxin, so it is easy to lose the recovery rate in the process of target substance adsorption and impurity elution. Therefore, a PRIME HLB column was selected as the purification column in this experiment.

### Matrix effect

**Figure 3:**
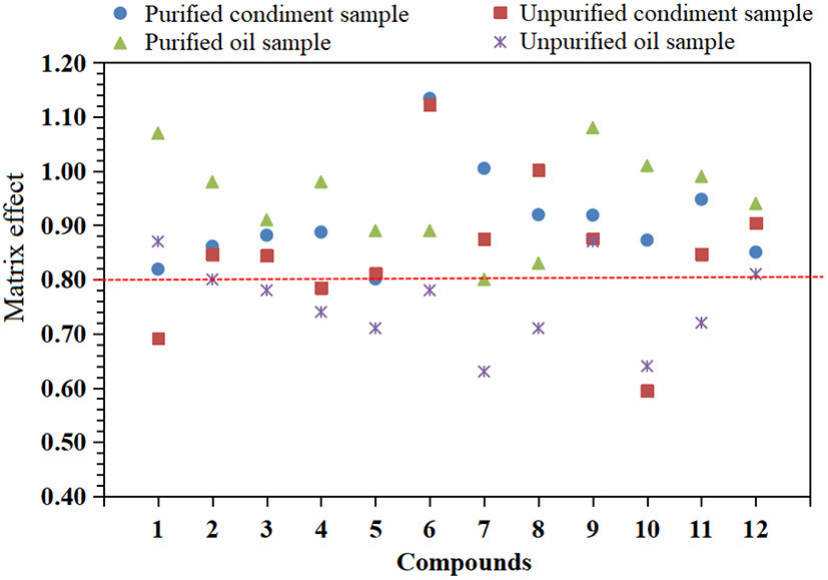
Matrix effects of 12 mycotoxins in purified and unpurified condiments and oil samples.

The matrix is a coextraction interfering substance other than the measured substance in the sample that often competes with the target compound for ionization, has significant interference with the analysis of the measured substance, and affects the accuracy of the determination results (29). These interferences and influences are called the matrix effect. The matrix effect is ion inhibition or ion enhancement. The existence of the matrix effect will affect the accuracy of the determination results (30, 31). The standard solution (analyte concentration is 10 μg/L) was prepared from the matrix extracts of edible oil and condiment blank samples without 12 mycotoxins. The matrix effect (ME) was evaluated by the ratio of the two with reference to the standard solution of the same concentration prepared by pure solvent, that is, the formula ME = B/A, where A and B represent the peak areas of analytes in pure solvent and blank sample matrix solution, respectively. If ME < 0.8, it indicates that the matrix has a significant inhibition on the response of analytes; if ME > 1.2, it indicates that the matrix will significantly enhance the response of analytes; if 0.8 ≤ ME ≤ 1.2, it indicates that the matrix effect is not significant (23, 32). The experimental results are shown in Figure 3. After purification by PRiME HLB column, the matrix effect of 12 mycotoxins in the two matrices is between 0.80 and 1.13 (see Table 2), and when it is not purified, the matrix effect is between 0.64 and 1.08. Some compounds have obvious matrix inhibition, which shows that purification by PRiME HLB column can obviously reduce the matrix effect, which is beneficial to reduce matrix interference and improve accuracy.

### Method validation

#### Linear relationship, LOD and LOQ

In this method, pure solvent was used to dilute the standard solution step by step to the lowest concentration that could be detected by the instrument, and the samples were injected repeatedly. According to the standard deviation of the test results, the detection limit (LOD, S/N ≥ 3) was determined by three times the signal-to-noise ratio, and the quantification limit (LOQ, S/N ≥ 10) was determined by 10 times the signal-to-noise ratio (33, 34). Regression analysis was carried out with the peak area (y) of the exact mass-to-charge ratio of the target parent ion as the ordinate and the compound concentration (x, μg/L) as the abscissa, and the linear regression equation, linear range and detection limit of each compound were obtained, as shown in Table 3. The peak area of 12 mycotoxins showed a good linear relationship with their mass concentrations; the determination coefficient *R*^2^ was > 0.998, the detection limit ranged from 0.12 to 1.2 μg/L, and the quantitative limit ranged from 0.4 to 4.0 μg/L, which indicated that the method had good sensitivity. The applied regulatory levels and standards of mycotoxins vary in the different regions of the world. The current maximum levels for aflatoxins set by the European Commission (EC) are 2 μg/kg for AFB1 and 4 μg/kg for total aflatoxins in various foods. These are to be extended to cover spices with limits of 5 and 10 μg/kg for AFB1 and total aflatoxins, respectively (35). Apart from aflatoxins, the maximum levels for OTA, DON, ZEN, and FBs (sum of FB1 and FB2) in various foods are also stipulated in Commission Regulation (EC) No 1881/2006 and in its amendments (36). The sensitivity parameters in this developed method are adequate to the legal limit requirements.

**Table 3:** Matrix effect, linear ranges, regression equations, determination coefficients, LODs, and LOQs for 12 mycotoxins.

**Compound**	**Matrix effect**		**Linear equation**	***R^2^***	**Linear range (μg/L)**	**LOD** **(μg/L)**	**LOQ** **(μg/L)**
	**Soy sauce and bean sauce**	**Oils**					
OTA	0.82	1.07	y =2.17641 × 10^5^x−1.57572 × 10^5^	0.99922	2–100	1.2	4.0
OTB	0.86	0.98	y = 5.27069 × 10^5^x−52541.489	0.99969	1–100	0.6	2.0
AFB1	0.88	0.91	y = 3.58221 × 10^6^x−1.30582 × 10^6^	0.99969	0.2–100	0.12	0.4
AFB2	0.89	0.98	y = 3.39177 × 10^6^x−1.11143 × 10^6^	0.99954	0.2–100	0.12	0.4
AFG1	0.80	0.89	y = 2.70403 × 10^6^x−3.11662 × 10^6^	0.99931	0.2–100	0.12	0.4
AFG2	1.13	0.89	y =2.32422 × 10^6^x−1.26201 × 10^6^	0.99974	0.2–100	0.12	0.4
HT-2	1.01	0.80	y = 2.51552 × 10^5^x−2.35324 × 10^5^	0.99969	2–100	1.2	4.0
ST	0.92	0.83	y =1.61012 × 10^6^x−1.23751 × 10^6^	0.99953	0.5–100	0.3	1.0
DAS	0.92	1.08	y = 7.06211 × 10^5^x + 1.81285 × 10^5^	0.99907	1–100	0.6	2.0
PA	0.87	1.01	y = 4.55992 × 10^5^x - 8.02080 × 10^5^	0.99947	2–100	1.2	4.0
MA	0.95	0.99	y = 4.97538 × 10^5^x + 4.86495 × 10^5^	0.99892	1–100	0.6	2.0
CIT	0.85	0.94	y = 2.65245 × 10^5^x −5.99355 × 10^5^	0.99914	2–100	1.2	4.0

#### Recovery and precision

The blank substrate samples of edible oil and condiment without the target substance to be tested were selected for the standard addition recovery experiment. Standard solutions with three concentration levels of LOQ, 2× LOQ, and 10 × LOQ were added, and six parallel samples were made for each standard addition concentration. The recovery rate was calculated, and the results are shown in Table 3. Table 4 shows that the average recovery rate of 12 mycotoxins in soy sauce and bean sauce is between 78.4 and 106.8%, and the precision RSD (*n* = 6) is between 1.2% and 9.7%. The average recoveries in edible oil ranged from 78.3 to 115.6%, and the precision RSD (*n* = 6) ranged from 0.9 to 8.6%.

**Table 4:** Recoveries and RSDs (*n* = 6) of blank samples fortified with 12 mycotoxins.

**Sample**	**Compounds**	**Spiked level**					
		**1 LOQ**		**2 LOQ**		**10 LOQ**	
		**Recovery** **(%)**	**RSD** **(%, *n*=6)**	**Recovery** **(%)**	**RSD** **(%, *n*=6)**	**Recovery** **(%)**	**RSD** **(%, *n*=6)**
Soy sauce and bean sauce	OTA	85.8	3.4	94.6	3.8	91.3	8.5
	OTB	78.4	3.3	106.2	2.3	102.4	7.6
	AFB1	95.6	1.7	97.2	6.4	89.2	3.5
	AFB2	94.8	2.6	100.5	7.6	90.6	2.9
	AFG1	80.4	6.3	95.4	3.5	86.1	1.9
	AFG2	99.0	3.9	106.8	1.3	99.5	5.4
	HT-2	82.9	7.9	89.4	2.6	80.3	4.8
	ST	82.6	1.5	104.2	9.7	100.8	8.1
	DAS	95.7	4.5	106.2	7.1	97.8	3.8
	PA	94.1	2.7	91.7	1.2	87.6	2.6
	MA	92.1	6.2	95.1	4.1	105.2	1.6
	CIT	94.8	3.2	99.3	2.9	97.3	7.9
Oils	OTA	97.0	1.3	93.3	5.1	101.3	2.2
	OTB	84.6	2.8	115.6	4.8	106.5	1.3
	AFB1	99.4	1.5	98.7	3.4	93.3	2.4
	AFB2	103.2	4.6	98.4	6.6	94.8	7.4
	AFG1	93.3	1.7	102.0	6.8	92.5	3.9
	AFG2	101.6	1.4	105.3	4.0	93.8	3.5
	HT-2	94.9	6.5	96.3	0.9	94.5	2.8
	ST	81.3	2.0	81.9	3.9	78.3	6.3
	DAS	93.9	4.1	100.4	5.1	97.6	3.9
	PA	85.5	8.6	102.1	3.1	89.8	6.1
	MA	87.4	7.7	95.3	6.6	101.8	1.2
	CIT	89.3	7.4	93.8	3.3	84.3	7.5

### Actual sample analysis

AFB2 was detected in 4 soy sauce and bean sauce samples (S2, S5, S11, and S13) under optimized experimental conditions with contents of 1.8–22.1 μg/kg (Table 5). AFB1 was detected in 6 edible oil samples (S15, S16, S18, S20, S23, S24) with contents ranging from 0.9 to 4.7 μg/kg. Sterigmatocystin was detected in S3 and S15 with contents of 2.9 and 2.1 μg/kg, respectively. Mycophenolic acid was detected in S3 at a concentration of 7.5 μg/kg. It could be inferred from the data that AFB2 is easily produced in soy sauce and bean sauce, and AFB1 is easily produced in edible oil. AFBs represent a global public health issue, as they are responsible for significant adverse health issues affecting consumers worldwide. AFB1, due to its toxic, mutagenic, immunotoxic, teratogenic, and carcinogenic effects on humans and animals, is classified as a group 1 carcinogen in the International Agency for Research on Cancer (IARC) classification of carcinogenic substances (37). To avoid or minimize health concerns, the EU has also established maximum tolerable limits for AFs in chillies as 10 μg/kg for total and 5 μg/kg for AFB1 (38). Therefore, the control of AFBs, especially AFB1 and AFB2, is particularly crucial due to their high contents in soy sauce, bean sauce and edible oil. Moreover, the need for normative updates regarding legal limits for sterigmatocystin and mycophenolic acid should be considered based on their biological toxicity, as they were detected in several samples.

**Table 5:** Concentrations of mycotoxins detected in the condiments and oils.

**Sample No**.	**Sample information**	**Concentrations (μg/kg)**	**Sample No**.	**Sample information**	**Concentrations (μg/kg)**
S1	Corn oil	ND^*^	S13	bean sauce	AFB2 (7.3)
S2	Light soy sauce	AFB2 (12.8)	S14	Rapeseed oil	ND
S3	Sesame oil	ST (2.9) MA (7.5)	S15	Peanut oil	AFB1 (1.0) ST (2.1)
S4	Oyster sauce	ND	S16	Peanut oil	AFB1 (1.0)
S5	Light soy sauce	AFB2 (22.1)	S17	Corn oil	ND
S6	Dark soy sauce	ND	S18	Peanut oil	AFB1 (1.4)
S7	Chili sauce	ND	S19	Rapeseed oil	ND
S8	Oyster sauce	ND	S20	Peanut oil	AFB1 (0.9)
S9	Dark soy sauce	ND	S21	Sesame oil	ND
S10	Dark soy sauce	ND	S22	Zanthoxylum oil	ND
S11	Oyster sauce	AFB2 (1.8)	S23	Peanut oil	AFB1 (1.0)
S12	Sweet bean sauce	ND	S24	Peanut oil	AFB1 (4.7)

* ND, Not detectable.

### Comparison with other reported methods

The comparison of this established method with other reported methods for the determination of mycotoxins in oil and sauce samples is summarized in Table 6. Table 6 clearly shows that HPLC and liquid chromatography with tandem mass spectrometry (LC–MS/MS) are the most commonly used methods for the determination of mycotoxins in separate matrices (38–41, 43, 44). For the pretreatment method, the QuEChERS procedure (quick, easy, cheap, effective, rugged and safe) seems to be the most commonly used technique (9, 39, 43–46). However, all these methods listed in Table 6 are suitable for the determination of several mycotoxins in either vegetable oil samples or sauce samples. The method established in our work can be used for the analysis of 12 mycotoxins in not only edible oil samples but also soy sauce and bean sauce samples. The PRiME HLB solid phase extraction combined with HPLC-Orbitrap HRMS developed in the present work shows rapid extraction time as well as favorable linearity, LODs, recoveries and RSDs, which are comparable or superior in comparison with other analytical methods. In addition, this is also the first study to develop an accurate method for the determination of sterigmatocystin (ST), diacetoxyscirpenol (DAS), penicillic acid (PA), mycophenolic acid (MA), and citreoviridin (CIT) in foodstuffs.

**Table 6:** Comparison with other methods reported in the literature^*^.

**Mycotoxins**	**Matrix**	**Instrumental method**	**Pretreatment method**	**LODs (μg/L)**	**Recoveries (%)**	**RSDs (%)**	**Reference**
4 mycotoxins (AOH, AME, TEN, and TeA)	tomato sauce	LC–MS/MS	QuEChERS	1.0–80	98.8–108.9	<10	(39)
5 mycotoxins (AFB1, AFB2, AFG1, AFG2, and OTA)	chili sauce	HPLC-FLD	Solvent extraction and IAC clean-up	0.05–0.1	86–93	6–15	(38)
5 mycotoxins (AFB1, AFB2, AFG1, AFG2, and OTA)	soybean paste	HPLC-FLD	Solvent extraction and IAC clean-up	0.01–0.2	/	/	(40)
6 mycotoxins (AFB1, AFB2, AFG1, AFG2, α-ZOL, and ZEA)	edible vegetable oil	UHPLC-QqQ-MS/MS	QuEChERS procedure	0.5–1.0	87.5–119.4	<20	(9)
4 mycotoxins (AFB1, AFB2, AFG1, and AFG2)	bean sauce	HPLC-UV	monolithic column based on covalent cross-linked polymer gels	0.08–0.2	76.1–113	1.1–9.6	(41)
6 mycotoxins (α-ZOL, β-ZOL, α-ZAL, β-ZAL, ZON, and ZAN)	edible vegetable oil	GC-QqQ MS	gel permeation chromatography	0.01–0.06	80.3–96.5	<11.6	(42)
9 mycotoxins (AFB1, AFB2, AFG1, AFG2, BEA, OTA, ZEA, FB1, and FB2)	vegetable oil	LC–MS/MS	QuEChERS-based procedure	0.02–14.66	70–120	<30	(43)
16 mycotoxins (α-ZAL, ZON, DON, β-ZAL, β-ZOL, α-ZOL, OTA, T-2,3-Ac-DON, 15-Ac-DON, AFB1, AFB2, AFG1, AFG2, AFM1, and AFM2)	vegetable oil	LC–MS/MS	QuEChERS-based extraction	0.04–2.9	72.8–105.8	<7	(44)
12 mycotoxins (AFB1, AFB2, AFG1, AFG2, OTA, OTB, HT-2, ST, DAS, PA, MA, and CIT)	edible oil, soy sauce and bean sauce	HPLC-Orbitrap HRMS	PRiME HLB solid phase extraction	0.12–1.2	78.3–115.6	0.9–9.7	This work

* Abbreviations: GC-QqQ MS, Gas chromatography-triple quadrupole-mass spectrometry; HPLC-UV, high-performance liquid chromatography-ultraviolet detection; UHPLC-QqQ-MS/MS, ultrahigh-performance liquid chromatography-triple quadrupole tandem mass spectrometry; HPLC-Orbitrap HRMS, high-performance liquid chromatography-tandem Orbitrap high-resolution mass spectrometry; LC–MS/MS, liquid chromatography with tandem mass spectrometry.

AFB1, Aflatoxin B1; AFB2, aflatoxin B2; AFG1, aflatoxin G1; AFG2, aflatoxin G2, AFM1, aflatoxin M1, AFM2, aflatoxin M2, OTA, ochratoxin A, ZON, zearalenone, ZAN, zearalanone, DON, deoxynivalenol, α-ZAL, α-zearalanol, β-ZAL, β-zearalanol, β-ZOL β-zearalenol, α-ZOL, α-zearalenol, T-2, T-2 toxin, HT-2, HT-2 toxin, 3-Ac-DON, 3-acetyldeoxynivalenol, 15-Ac-DON, 15-acetyldeoxynivalenol, FB1, fumonisin B1, FB2, fumonisin B2, BEA, beauvericin, ST, sterigmatocystin, DAS, diacetoxyscirpenol, PA, penicillic acid, MA, mycophenolic acid, CIT, citreoviridin, AOH, alternariol.

## Conclusion

Aiming at the present situation that edible oil, soy sauce and bean sauce are easily contaminated by many mycotoxins simultaneously, a new impurity adsorption purification technology was adopted, and a simultaneous determination method for rapid screening and quantitative determination of 12 mycotoxins by HPLC-Orbitrap HRMS was established. The pretreatment operation is simple and rapid. The method realizes simultaneous analysis and detection of various mycotoxins, with high stability and sensitivity, strong specificity and good reproducibility. The LODs of the 12 mycotoxins were 0.12–1.2 μg/L. The average recoveries in edible oil, soy sauce and bean sauce were 78.3–115.6% with RSDs of 0.9–9.7%. Therefore, the developed method can be used as a quantitative method for the determination of various mycotoxins in edible oils, soy sauce and bean sauce.

## Data availability statement

The original contributions presented in the study are included in the article/Supplementary material, further inquiries can be directed to the corresponding author/s.

## Author contributions

DL prepared and wrote the manuscript. JG, JC, and ML corrected, revised, and improved the manuscript. CZ and YX, modified the Tables and Figures in the manuscript. HD and XX designed the study, supervised, reviewed, and finalized the manuscript. All authors contributed to the article and approved the submitted version.

## Funding

This work was financially supported by Guangdong Provincial Key R&D Programme (No. 2021B0707060001), project (No. 2020PT01) Science and Technology Project of Chaozhou Branch of Chemistry and Chemical Engineering Guangdong Laboratory (No. HJL202202B007), and Characteristic Innovation Project of Universities in Guangdong province (2022KTSCX058).

## Conflict of interest

The authors declare that the research was conducted in the absence of any commercial or financial relationships that could be construed as a potential conflict of interest.

## Publisher's note

All claims expressed in this article are solely those of the authors and do not necessarily represent those of their affiliated organizations, or those of the publisher, the editors and the reviewers. Any product that may be evaluated in this article, or claim that may be made by its manufacturer, is not guaranteed or endorsed by the publisher.
